# Why is it so difficult to access emergency contraceptive pills in Japan?

**DOI:** 10.1016/j.lanwpc.2021.100095

**Published:** 2021-01-28

**Authors:** Sumire Sorano, Sakiko Emmi, Chris Smith

**Affiliations:** a School of Tropical Medicine and Global Health, Nagasaki University, 1-14 Bunkyomachi, Nagasaki 852-8521, Japan; b Faculty of Infectious and Tropical Diseases, Department of Clinical Research, London School of Hygiene & Tropical Medicine, London, UK; c Department of Social Psychiatry and Mental Health, Faculty of Medicine, University of Tsukuba, Japan; d Citizen’s Initiative for Pharmaceutical Access To Emergency Contraception (CIPATEC)

Since 8th October when the government announced that a discussion to approve emergency contraceptive pills (ECPs) for non-prescription provision will be restarted in Japan [1], heated debates over the issue have reignited in the mainstream and social media. Why has improving access to ECPs become so controversial in Japan?

Currently, access to ECPs in Japan requires a medical prescription and a pill costs around 6000 to 20,000 yen (55–190 USD), which is unacceptably expensive for many women. This is because contraception is not covered by national health insurance and the price is set at the discretion of each medical facility. Currently only 3% of hospitals and clinics declare that they provide consultation for ECPs and of those, only a few extended opening hours [2]. Concerns over unintended pregnancy increased amid the COVID-19 pandemic where consultations for unintended pregnancy and sexual violence increased significantly [3].

The World Health Organization (WHO) states that it is a right of all women and girls at risk of an unintended pregnancy to access emergency contraception. Considering its safety and higher effectiveness in earlier use after unprotected sex, the WHO and the International Federation of Gynecology and Obstetrics (FIGO) recommend non-prescription provision of ECPs [4,5]. Currently, 19 countries allow direct access to ECPs over the counter and 76 countries from a pharmacist without a prescription.

In response to public demand for over-the-counter (OTC) use, the MHLW considered approval of non-prescription provision of ECPs in 2017 [6]. Despite receiving overwhelming public support, the plan did not pass due to concerns raised by the review committee including that users might not have enough “literacy” to adequately use it and that it might lead to “misuse” or “abuse” [6]. Concerns were raised mainly by the Japanese Association of Obstetricians and Gynecologists (JAOG) [6].

First is a safety concern. The review committee discussed that it is necessary for physicians with “specialized knowledge of reproductive endocrinology” to determine who is eligible and to follow-up after use because users might not be able to notice pregnancy in case of ECP failure [6]. Is the safety concern substantial enough to necessitate specialist screening and regular follow-up? Although ECPs are not indicated for a woman who is already pregnant, current evidence suggests that ECPs do not harm a fetus [5]. The WHO and major societies of obstetricians and gynaecologists do not recommend provider screening or regular follow-up because ECPs are safe for all women and should not be delayed for screening and pregnancy test [4,5,7].

Secondly, there is an argument that increased access to ECPs might discourage condom use and increase sexually transmitted infections. However, there is no evidence that providing advance ECPs to adolescents is associated with more unprotected intercourse or less condom or hormonal contraception use [8].

The third concern is about abuse or misuse. In 2019, the JAOG made a written statement [9] that “women seeking emergency contraceptives, or people behind them, may be involved in the sex industry or a criminal organization who might transfer drugs to other”. They continue that thus it is desirable to limit prescription to one set at a time and make women take the pill on the spot. This recommendation of directly observed use based on suspicion that women might abuse the medication is unbearable and problematic. This argument is clearly going against international evidence-based recommendations by WHO and FIGO that encourage advance supply since studies show that women given ECPs beforehand were more likely to use them when needed and to take them sooner [4,5].

Historically, Japan was the only United Nations member prohibiting the use of low-dose oral contraceptives (OCs) until 1999 when approval was given after approximately 44 years of debate [10,11]. Current discussion of ECPs remind us of the debate over OCs approval where gender-biased and paternalistic arguments were made such as “OCs would deteriorate sexual morality or spread sexually transmitted infections” [10].

This is an urgent issue since there are women on a day-to-day basis with immediate need of this essential medication. A petition for approving ECPs for non-prescription provision has gained more than 107,000 signatures to date up (15 Nov 2020) [12]. Policy makers and specialist advisory groups should listen to the voices of people and to make an evidence-based decision.

## Contributions

SE designed the overall structure of manuscript. SS analyzed the data and drafted the early version of the manuscript. CS gave comments on the earlier versions of the manuscript. All authors edited the manuscript and approved the final version.

## Declaration of Competing Interest

The authors declare no conflicts of interest.

## Role of funding source

SS is supported by WISE Program (Doctoral Program for World-leading Innovative & Smart Education) of Ministry of Education, Culture, Sports, Science and Technology. The funders had no role in study design, data collection and analysis, decision to publish, or preparation of the manuscript.
